# NF-κB inhibition alleviates carbon tetrachloride-induced liver fibrosis via suppression of activated hepatic stellate cells

**DOI:** 10.3892/etm.2014.1682

**Published:** 2014-04-14

**Authors:** FEI WANG, SHUYUAN LIU, TAIPING DU, HAO CHEN, ZHIYONG LI, JINGWANG YAN

**Affiliations:** 1 Department of General Surgery Ward 1, Xinxiang Central Hospital, Xinxiang, Henan 453000, P.R. China; 2 Department of Infectious Diseases, Third Affiliated Hospital of Xinxiang Medical College, Xinxiang, Henan 453000, P.R. China

**Keywords:** nuclear factor-κB, BAY-11-7082, liver fibrosis, carbon tetrachloride

## Abstract

An effective treatment for hepatic fibrosis is not available clinically. Nuclear factor (NF)-κB plays a central role in inflammation and fibrosis. The aim of the present study was to investigate the effect of an NF-κB inhibitor, BAY-11–7082 (BAY), on mouse liver fibrosis. The effects of BAY on hepatic stellate cell (HSC) activation were measured in the lipopolysaccharide-activated rat HSC-T6 cell line. In addition, the therapeutic effect of BAY was studied *in vivo* using a model of hepatic fibrosis induced by carbon tetrachloride (CCl_4_) in mice. BAY effectively decreased the cell viability of activated HSC-T6 cells and suppressed HSC-T6 activation by downregulating the expression of collagen I and α-smooth muscle actin. BAY significantly inhibited the phosphorylation of phosphatidylinositol 3-kinase (PI3K) and serine/threonine kinase-protein kinase B (Akt) in activated HSC-T6 cells. In addition, administration of BAY attenuated mouse liver fibrosis induced by CCl_4_, as shown by histology and the expression of profibrogenic markers. BAY also significantly decreased the levels of serum alanine aminotransferase in this model of hepatic fibrosis. Therefore, the results of the present study demonstrate that BAY attenuates liver fibrosis by blocking PI3K and Akt phosphorylation in activated HSCs. Thus, BAY demonstrates therapeutic potential as a treatment for hepatic fibrosis.

## Introduction

Liver fibrosis is a common consequence of various chronic liver diseases and the underlying pathology represents the common response of the liver to toxicity, infection or metabolism (1–3). Hepatic fibrosis, characterized by excess deposition of extracellular matrix proteins, is traditionally viewed as an irreversible pathological process involving multiple signaling pathways (4,5). With protracted damage, fibrosis progresses into excessive scarring and organ damage, including liver cirrhosis. However, recent evidence has indicated that liver fibrosis may be dynamic and bidirectional, involving progression and regression (6), offering an opportunity of therapeutic intervention to halt or reverse fibrosis. To date, antifibrotic treatment represents an unconquered area for drug development, with enormous potential but also high risks (7).

During liver fibrosis, hepatic stellate cells (HSCs) are primarily activated by transforming growth factor-β, in addition to other profibrotic cytokines. Upon activation, HSCs proliferate and differentiate into myofibroblasts which secrete several extracellular matrix constituents, including collagens (8,9). Activated HSCs are the key cells involved in the progression of liver fibrosis (10). Nuclear factor (NF)-κB is a heterodimeric transcription factor that plays a central role in the pathogenesis of a wide variety of conditions affecting the liver, including hepatitis and fibrosis (11). Although the role of NF-κB signaling in the liver has been extensively explored, further studies of NF-κB signaling in liver fibrosis are required to promote translational application in liver disease. Thus, in the present study, the effect of an NF-κB inhibitor, BAY-11–7082 (BAY), was investigated in carbon tetrachloride (CCl_4_)-induced mouse model of liver fibrosis.

## Materials and methods

### Cell culture

HSC-T6 cells, an immortalized rat HSC cell line transfected with the SV40 large T-antigen containing a Rous sarcoma virus promoter, were purchased from the Cancer Institute and Hospital (Chinese Academy of Medical Sciences, Beijing, China). The Chang liver cell line (American Type Culture Collection, Manassas, VA, USA) was used as a normal human cell line derived from normal liver tissue (12). The cells were cultured in Dulbecco’s modified Eagle’s medium supplemented with 10% fetal bovine serum, penicillin G and streptomycin at 37°C. Cell passage of the cultures was performed every 3 days and the cells were plated in culture dishes at a density of 1×10^6^ cells. Next, the cells were treated with various concentrations (6.25, 12.5, 25 and 50 μM) of BAY 1 h prior to stimulation with 1 μg/ml lipopolysaccharide (LPS) for 24 h. The study was approved by the ethics committee of Xinxiang Central Hospital (Xinxiang, China).

### MTT assay

Cell viability was evaluated using an MTT assay. HSC-T6 and Chang liver cells were independently seeded in 96-well plates (1×10^4^ cells per well). HSC-T6 cells were treated with BAY (CAS19542-67-7; Cayman Chemical Co., Ann Arbor, MI, USA) and 1 μg/ml LPS, while normal Chang liver cells were treated with BAY. Following treatment with BAY and/or LPS for 24 h, 5 mg/ml MTT solution was added and the cells were incubated for an additional 3 h. The results were obtained as absorbance measurements at 490 nm using an ELISA microplate reader (3550, Bio-Rad, Hercules, CA, USA).

### Animals

C57BL/6 male mice were maintained in conditions according to the guidelines of the National Institutes of Health Guide for the Care and Use of Laboratory Animals (Institute of Laboratory Animal Resources, 1996). Mice were purchased and housed in a barrier facility. At the end of each experiment, the animals were sacrificed with CO_2_ following anaesthesia. The animals were also weighed and blood samples were collected. Whole livers were harvested and weighed. Liver samples were harvested from the two liver lobes to reduce sampling variability among the experimental and control mice.

### CCl_4_-induced mouse fibrosis model

The fibrosis model was generated using CCl_4_ (Sigma-Aldrich, St. Louis, MO, USA) dissolved at a concentration of 20% in olive oil. Intraperitoneal injections of 1 ml pure CCl_4_/kg body weight (dissolved at a concentration of 20% in olive oil) were administered twice a week for 6 weeks (13).

### Treatment protocols

The dosage of BAY was determined according to previous studies (14,15). BAY was dissolved in 10% dimethyl sulfoxide (DMSO)/phosphate-buffered saline (PBS). The treatment group received intraperitoneal injections of 5 mg/kg BAY three times a week as previously described, whereas the control groups received the vehicle only. Mice were randomly divided into three groups (n=12). Group 1 received 10% DMSO/PBS treatment, while group 2 received CCl_4_ only. Group 3 mice received 10 mg/kg BAY and CCl_4_. At the end of the first week, the CCl_4_-injected mice were intraperitoneally administered 10 mg/kg BAY three times a week for 6 weeks.

Animals were sacrificed 24 h following the last injection and blood samples were collected. Serum was then separated by centrifugation at 800 × g for 10 min at 4°C. The liver of each mouse was removed immediately and stored at −80°C for subsequent analysis.

### Measurement of serum alanine aminotransferase (ALT)

Mouse sera were collected and enzyme ALT levels were measured using the serum biochemical analyzers Ektachem DTSC-II analyzer (Eastman Kodak, Rochester, NY, USA) and Hitachi autoanalyzer (Tokyo, Japan), according to the manufacturer’s instructions.

### Histopathological analysis

Mice were sacrificed at the end of week 6, 24 h after the last injection). Liver samples from the left lateral and median lobes were separated and fixed in 10% neutral buffered formalin. The samples were then embedded in paraffin, sectioned (5 μm) and stained with Sirus red (Vector Laboratories, Inc., Burlingame, CA, USA) for general observations. A certified histopathologist was blinded to the group distribution throughout the analysis.

### Western blotting

Equal amounts of protein were resolved by 12.5% SDS-PAGE and immobilized on polyvinylidene fluoride membranes by wet transfer. Following blocking for 30 min with 5% non-fat dry milk in Tris-buffered saline-Tween 20, the membranes were exposed overnight at 4°C to primary antibodies. This was followed by incubation for 2 h at room temperature with the corresponding horseradish peroxidase (HRP)-conjugated secondary antibodies (Vector Laboratories, Inc.). Equal protein loading was corrected by the immunoblotting of β-actin. Immunoreactive proteins were visualized using a chemiluminescent HRP antibody detection reagent (Denville Scientific, Inc., South Plainfield, NJ, USA) and exposure to X-ray film (Eastman Kodak). Band density was analyzed using ImageJ software. The primary antibodies anti-p-phosphatidylinositol 3-kinase (PI3K)/PI3K, anti-p-Akt/Akt, anti-collagen I, anti-α smooth muscle actin (SMA; 1:1,000) and anti-β-actin antibody (1:2,500), were purchased from Santa Cruz Biotechnology, Inc. (Santa Cruz, CA, USA).

### Hydroxyproline Measurement

Liver tissue was homogenized in ice-cold distilled water (900 μl) using a Power Gen homogenizer (Fisher). Subsequently, 125 μl of 50% (wt/vol) trichloroacetic acid was added, and the homogenates were incubated further on ice for 20 min. Precipitated pellets were hydrolyzed for 18 h at 110°C in 6 N HCL. After hydrolysis, the samples were filtered and neutralized with 10 N NaOH, and the hydrolysates were oxidized with Chloramine-T (Sigma) for 25 min at room temperature. The reaction mixture then was incubated in Ehrlich’s perchloric acid solution at 65°C for 20 min and cooled to room temperature. Sample absorbance was measured at 560 nm in duplicate. Purified hydroxyproline (Sigma) was used to set a standard. Hydroxyproline content was expressed as microgram of hydroxyproline per g liver.

### Statistical analysis

Data are expressed as mean ± SD. Animal experiments were performed with 12 animals in each treatment and control group. All *in vitro* data is reported as the result of three independent experiments, including three replicates per experiment. Statistical analysis was performed using SPSS 17.0 software (SPSS, Inc., Chicago, IL, USA) and statistical differences between the groups were analyzed using the Student’s T test or one-way analysis of variance. P<0.05 was considered to indicate a statistically significant difference.

## Results

### Effect of BAY on cell viability

**Figure 1:**
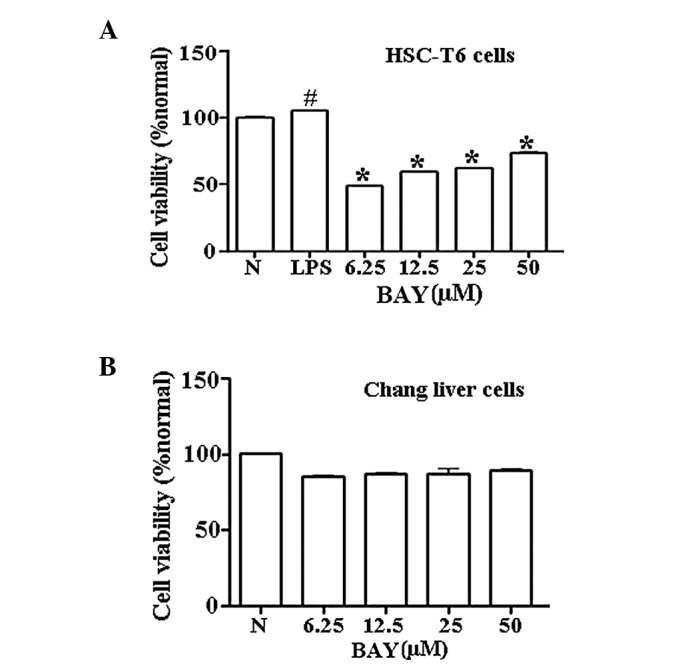
Effect of BAY on cell viability. Cell viability of (A) HSC-T6 cells treated with various concentrations of BAY (6.25–50 μM) after 24-h LPS induction and (B) normal control human Chang liver cells after treatment with BAY for 24 h, as determined by an MTT assay. Data are expressed as mean ± SD. ^#^P<0.01, vs. normal; ^*^P<0.01, vs. LPS-activated HSC-T6 cells. N, normal; LPS, lipopolysaccharide; HSC, hepatic stellate cell; BAY, BAY-11–7082.

Various concentrations of BAY (6.25–50 μM) significantly reduced the cell viability of HSC-T6 cells in a dose-dependent manner within 24 h following LPS stimulation (Fig. 1A). To determine whether BAY was cytotoxic to normal hepatocytes, normal human Chang liver cells were selected as a normal control to test the cell viability in the presence of various concentrations of BAY. At concentrations between 6.25 and 50 μM, BAY exhibited insignificant toxicity in normal Chang liver cells (Fig. 1B).

### Effect of BAY on the protein expression of collagen I and α-SMA

**Figure 2:**
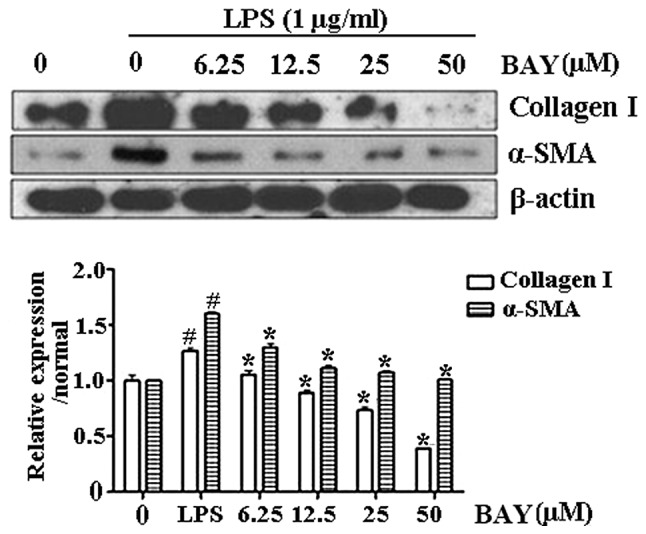
Effect of BAY on collagen I and α-SMA expression *in vitro*. HSC-T6 cells were pretreated with the indicated concentrations of BAY 1 h prior to incubation with 1 μg/ml LPS for 24 h. Collagen I and α-SMA proteins were detected by western blotting and β-actin was used as a internal control. Data were normalized against β-actin. ^#^P<0.01, vs. normal; ^*^P<0.01, vs. LPS-stimulated cells. LPS, lipopolysaccharide; HSC, hepatic stellate cell; BAY, BAY-11–7082; SMA, smooth muscle actin.

Activation of HSCs plays a central role in liver fibrosis and α-SMA is an established indicator of HSC activation (3). Collagen I is the principal collagen responsible for fibrosis and is generated by activated HSCs. The levels of α-SMA and collagen I were upregulated in LPS-activated HSC-T6 cells, indicating that HSCs were activated upon LPS administration. By contrast, BAY decreased the protein levels of α-SMA and collagen I in the LPS-treated cells (Fig. 2). These results demonstrated that BAY reduced HSC activation.

### Effect of BAY on LPS-induced phosphorylation of PI3K/Akt

**Figure 3:**
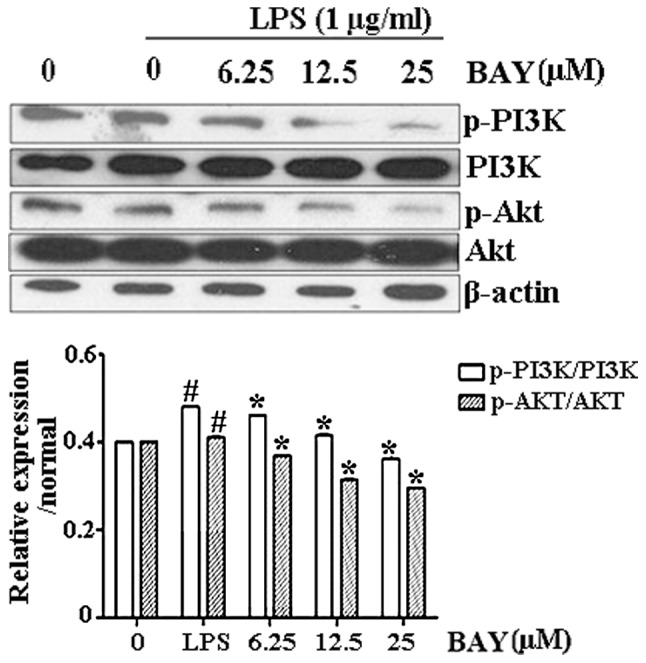
Effect of BAY on the phosphorylation of PI3K/Akt *in vitro*. HSC-T6 cells were pretreated with various concentrations of BAY for 1 h prior to stimulation with 1 μg/ml LPS for 24 h. Total and phosphorylated PI3K/Akt levels were detected by western blotting and β-actin was used as a loading control. Data were normalized against β-actin expression levels. ^#^P<0.01, vs. normal; ^*^P<0.01, vs. LPS-stimulated cells. LPS, lipopolysaccharide; HSC, hepatic stellate cell; BAY, BAY-11–7082; PI3K, phosphatidylinositol 3-kinase; Akt, protein kinase B.

To investigate the antifibrotic mechanism of BAY and the possible association with the PI3K/Akt signaling pathway, PI3K/Akt expression was observed in activated HSC-T6 cells. PI3K and Akt phosphorylation was upregulated following LPS stimulation; however, the phosphorylation levels of PI3K/Akt were significantly reduced by BAY treatment in a dose-dependent manner (Fig. 3).

### Effect of BAY on CCl_4_-induced hepatic fibrosis

**Figure 4:**
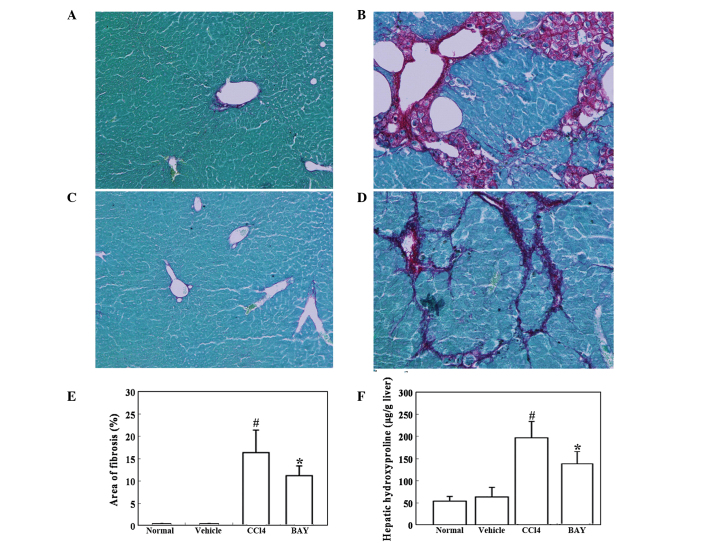
Effect of BAY on CCl_4_-induced liver fibrosis. Mice were intraperitoneally injected with 1 ml/kg CCl_4_ twice a week in combination with 5 mg/kg BAY three times a week for 6 weeks. (A–D) Liver fibrosis was detected by Sirius red staining. Representative micrographs of histology from (A) normal, (B) vehicle (10% DMSO/PBS), (C) CCl_4_ and (D) CCl_4_ + BAY treated mice (magnification, ×100). (E) Morphometrical analysis for evaluating the percentages of α-SMA-positive areas in 12 random fields. (F) Hepatic hydroxyproline was detected. All data are expressed as the mean ± SD of 12 mice. ^#^P<0.01, vs. control; ^*^P<0.01, vs. CCl_4_. BAY, BAY-11–7082; SMA, smooth muscle actin; CCl_4_, carbon tetrachloride; DMSO, dimethyl sulfoxide; PBS, phosphate-buffered saline.

Mouse hepatic fibrosis was determined using Sirius red staining. As expected, marked bridging fibrosis was observed in the mice treated with vehicle (Fig. 4B). BAY significantly attenuated the CCl_4_-induced liver fibrosis (Fig. 4D). Further analysis demonstrated that the area of hepatic fibrosis was significantly reduced in BAY and CCl_4_-treated mice compared with that in the mice treated with CCl_4_ alone (Fig. 4E). The effect of BAY on hepatic hydroxyproline, which is indicative of hepatic fibrosis, was then studied. CCl_4_ administration significantly increased the hepatic hydroxyproline content in the mice, while BAY administration significantly reduced this CCl_4_-induced increase in hepatic hydroxyproline content (Fig. 4F).

### Effect of BAY on serum ALT levels and expression of collagen I and α-SMA in CCl_4_-induced mouse liver injury

**Figure 5:**
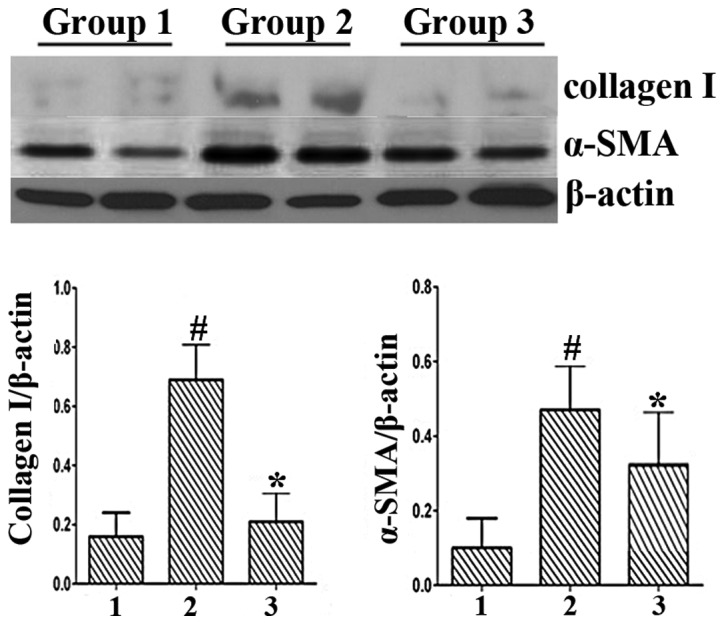
Effect of BAY on the protein expression levels of collagen I and α-SMA in CCl_4_-induced mouse liver injury. CCl_4_-induced liver injury revealed high expression levels of collagen I and α-SMA by western blotting. BAY decreased the protein expression levels of collagen I and α-SMA in the liver injury model. The *in vivo* data were consistent with the *in vitro* results. ^#^P<0.01, vs. control; ^*^P<0.01, vs. CCl_4_. BAY, BAY-11–7082; SMA, smooth muscle actin; CCl_4_, carbon tetrachloride.

Serum ALT levels were determined as an indicator of liver function and the ability of BAY to reduce serum ALT levels in CCl_4_-induced liver injury was investigated. ALT levels were significantly elevated in the CCl_4_ group (Group 1 vs. Group 2, 38.96±5.88 vs. 448.45±78.40 U/l; P<0.001). However, BAY treatment significantly attenuated the CCl_4_-induced increase in ALT levels (Group 2 vs. Group 3, 448.45±78.40 vs. 361.37±82.51 U/l; P<0.001). In addition, CCl_4_-induced liver injury revealed high expression levels of collagen I and α-SMA by western blotting (Fig. 5) and BAY was shown to decrease the protein expression levels of collagen I and α-SMA in the liver injury model. These *in vivo* results were consistent with the *in vitro* results.

## Discussion

Chronic inflammation and the associated regenerative wound-healing response are strongly associated with the development of fibrosis and cirrhosis (16). In the past decade, numerous inflammatory mediators have been shown to contribute to the progression of chronic liver disease, a number of which are targets or activators of NF-κB (17–20). Studies targeting this molecule as an appropriate therapeutic agent in various diseases are ongoing. However, it is necessary to demonstrate whether NF-κB antagonism effectively treats pre-existing hepatic fibrosis and the potential mechanism of action. In the present study, the NF-κB inhibitor, BAY, effectively suppressed HSC-T6 activation by downregulating the expression of collagen I and α-SMA. BAY also inhibited PI3K and Akt phosphorylation in activated HSC-T6 cells.

Kupffer cells contribute to HSC activation and liver fibrosis (21). Inhibition of NF-κB in Kupffer cells results in decreased liver fibrosis; however, the underlying mechanisms remain largely elusive (22). While the role of NF-κB activation in hepatocytes and Kupffer cells leading to liver fibrosis is not completely understood, there is growing evidence that NF-κB functions as a key mediator of fibrosis. A wide range of proinflammatory mediators activate NF-κB in HSCs, including LPS, tumor necrosis factor and interleukin-1β (23–26). In addition, HSCs activate NF-κB during culture activation (27) and in human and mouse models of liver fibrosis, as demonstrated by the presence of Ser 536-phosphorylated p65 (25). Notably, NF-κB activation is almost exclusively observed in HSCs, indicating that these cells are an important site of inflammation in a chronically injured and fibrotic liver (28). Notably, the results of the present study demonstrate that the administration of BAY attenuates liver fibrosis induced in mice by the administration of CCl_4_. BAY also significantly decreased the levels of serum ALT in the model mice.

PI3K is a key signaling molecule that controls numerous cellular functions (29). In the liver, PI3K activation promotes cytokine production and subsequent hepatocyte proliferation following partial hepatectomy (30). Hepatocyte-associated PI3K regulates hepatocyte growth by a process involving Akt activation (30). In the present study, fibrogenesis, which may be promoted by PI3K, was inhibited in association with reduced α-SMA expression and collagen production. PI3K/Akt signaling activation was also inhibited by BAY in LPS-treated HSCs and the fibrotic mouse liver. Inhibition of PI3K/Akt signaling may be strongly associated with the antifibrogenic effect.

In summary, the present study demonstrates that NF-κB signaling is activated in the pathogenesis of CCl_4_-induced hepatic fibrosis. BAY, an NF-κB inhibitor, inhibits CCl_4_-induced hepatic PI3K/Akt signaling activation. In addition, BAY attenuates CCl_4_-induced HSC activation and effectively alleviates CCl_4_-induced hepatic fibrosis in mice. Thus, NF-κB inhibition may have potential therapeutic value against hepatic fibrosis.
